# Preliminary Proteomic Study of the Porcine Pituitary Gland under Heat Stress

**DOI:** 10.3390/life14030366

**Published:** 2024-03-11

**Authors:** Qiu Zhou, Yuan Gao, Yin Li, Huili Xie, Xiaoxi Liu, Yanhong Yong, Youquan Li, Zhichao Yu, Xingbin Ma, Xianghong Ju

**Affiliations:** 1 Marine Medical Research and Development Centre, Shenzheng Institute, Guangdong Ocean University, Shenzheng 518120, China; 2112104098@stu.gdou.edu.cn (Q.Z.); 2112104078@stu.gdou.edu.cn (Y.G.); ly12181028@163.com (Y.L.); 2112104024@stu.gdou.edu.cn (H.X.); liuxiaoxi_06@163.com (X.L.); yongyanhong-007@163.com (Y.Y.); youquan-li@163.com (Y.L.); yujingmary@163.com (Z.Y.); mxb1984612@126.com (X.M.); 2 College of Coastal Agricultural Sciences, Guangdong Ocean University, Zhanjiang 524088, China

**Keywords:** heat stress, quantitative proteomics, pituitary gland, functional networks

## Abstract

Although numerous studies have shown that the hypothalamic–pituitary–adrenal axis plays a vital role in the response to environmental stress by mediating the production of a series of hormones, the mechanism underlying these effects has not been elucidated. This study used proteomics techniques to investigate the differentially expressed proteins (DEPs) in the pituitary glands of pigs and to elucidate the potential changes in the immune–neuroendocrine system under heat stress (HS). In total, 2517 peptides corresponding to 205 proteins were detected. A comparison of the expression patterns between HSs and healthy controls revealed 56 DEPs, of which 31 were upregulated and 25 were downregulated. Ingenuity pathway analysis (IPA) was used to reveal the subcellular characteristics, functional pathways, regulatory networks, and upstream regulators of the identified proteins. The results showed that these differentially expressed proteins were involved in intercellular communication, interactions, apoptosis, nervous system development, functions, abnormalities and other functions, and in the regulatory network. Moreover, the upstream regulators of the differentially expressed proteins were mainly transcriptional regulators, hormones, and cytokines. Thus, the functional network and pathway analyses could provide insights into the complexity and dynamics of HS–host interactions and may accelerate our understanding of the mechanisms underlying HS.

## 1. Introduction

With increasing global warming, heat stress has gained prominence as a factor increasing the risk of infectious diseases in pigs. In comparison with other animals, pigs exhibit high caloric metabolism, rapid fat deposition, and poor development of sweat glands. Thus, when the ambient temperature exceeds the upper limit of the thermal neutrality zone for pigs (16–22 °C for growing pigs), stress is more likely to occur [1]. Under heat stress, the metabolism, immune function, and growth performance of pigs are negatively affected [2,3,4]. Even moderate stress can enhance macrophage phagocytosis [5,6], NK cell activity [7], and antiviral infection [8], thereby playing an important role in immunosuppression and resulting in major economic losses in the swine industry. For instance, in the United States, the economic losses caused by heat stress in animal husbandry are worth at least several billion dollars each year [9].

After Besedovsky first proposed the concept of a neuroendocrine immune network in 1977 [10], a large number of studies have shown a bidirectional regulatory relationship between the neuroendocrine and immune systems [11,12]. The neuroendocrine system regulates the function of the immune system through neurotransmitters, hormones, cytokines secreted by nerve cells, and peripheral nerve synapses. Recent studies have shown that the secretion of hormones produced by the hypothalamic–pituitary–adrenal (HPA) axis is a major part of the neuroendocrine system [13] and plays an important role in health and disease by regulating immunoreactivity [14,15,16].

The HPA axis, sympathetic nerves, and adrenal medulla constitute the body’s stress system. Under the stimulation of stressors, the neurosecretory cells of the stress system are believed to produce neurohypophysis along the hypothalamic pituitary nerve bundles, reach the pituitary through the pituitary stalk, and promote the secretion of some hormone-releasing factors in the pituitary, inducing the target tissue to secrete specific hormones or factors to regulate the body and complete specific physiological activities [17]. Quinteiro-Filho et al. reported that acute heat stress activated HPA axis function, which accordingly increased the expression of serum corticosterone and decreased the expression of performance variables and the incidence of macrophage oxidative bursts, eventually leading to mild, multifocal acute enteritis [18]. As the major stress hormones in the HPA axis, glucocorticoids can cause apoptosis in immune cells, decrease lymphocyte proliferation, and impair dendritic cell (DC) function [19,20], thereby inducing immunosuppression in animals. In contrast, certain concentrations of corticosterone have also been shown to promote the secretion of TNF-α and IL-6 from macrophages and enhance phagocytosis [7,21].

Proteomics technology has gained importance in the postgenome era of life science research. For example, this technology was used to identify a new natural immune protein, H2A.1, which is significantly downregulated in the neutrophil membrane under immunosuppressive conditions [22]. Subsequent studies also identified a new mechanism by which neutrophils kill microorganisms [23]. Proteomic studies of milk mitochondrial membranes revealed that the Toll-like receptor (TLR)-2, TLR4, and CD14 signaling molecules are involved in breast mucosal immune processes [24]. These techniques were also used to identify new diagnostic methods for paratuberculosis [25], *Brucella* [26], *Pasteurella multocida* [27], and mycobacteria [28].

Under heat stress conditions, the hypothalamic–pituitary–adrenal axis is activated. As a central organ in the HPA axis, the pituitary gland is known to influence the function of the adrenal gland through the secretion of the adrenalotropic hormone, but the molecules that play a role in the regulation of heat stress by this organ are poorly understood. Thus, the present study aimed to investigate the changes in the proteins in the pituitary of pigs under heat stress.

## 2. Material and Methods

### 2.1. Animals and Housing

Six castrated Bama miniature pigs (*Sus scrofa domestica*) were obtained from a Bama Miniature Pigs Breeding Farm in the Guangxi Zhuang Autonomous Region of China. The six pigs (average weight, 30–40 kg) were randomly divided into a heat stress group (3 barrows, HS) and a control group (3 barrows, CC). The animals were allowed to acclimatize for one week before the start of the experiments. The control pigs were kept at a temperature of 28 ± 3 °C and a relative humidity of approximately 90%. The pigs in the heat stress group were kept at a temperature of 35 ± 1 °C and a relative humidity of approximately 90% in an artificial climate room for 7 days. All pigs were given access to water ad libitum. The full-price feed was obtained from Beijing Dabei Agricultural Technology (Beijing, China). Pigs were fed twice a day, once in the morning and once in the evening. All animal experiments were conducted in accordance with the guidelines provided by the National Research Council’s Guide for the Care and Use of Laboratory Animals.

### 2.2. Pituitary Gland Sample Collection

On the 7th day of heat stress, the pigs were sacrificed by carotid artery exsanguination. The pituitary tissue was removed, washed three times with sterilized saline, and then snap-frozen in liquid nitrogen for subsequent experiments.

### 2.3. Protein Extraction and Quantification

After removing the extra fat and connective tissue, the appropriate amount of the pituitary sample was weighed. The sample was eluted with 1 mL of phosphate-buffered saline (PBS) for 30 s and then centrifuged at 25,000× *g* for 20 min at 4 °C. After centrifugation, the supernatant was discarded, and the process was repeated twice. The sediment was redissolved in 500 μL of lysis solution (Lysis3), and 1 mM of phenylmethylsulfonyl fluoride (PMSF) and 2 mM of ethylenediaminetetraacetic acid (EDTA) were added to the solution. After 5 min, 10 mM of dithiothreitol (DTT) was added. The solution was ultrasonically disrupted for 15 min at 200 W and then centrifuged at 25,000× *g* for 20 min. The retained supernatant was treated with 10 mM of DTT at 56 °C for 1 h to open the disulfide bonds and then blocked using 55 mM of IAM for 45 min in a dark room for cysteine alkylation blocking. Subsequently, 1 mL of frozen acetone was added to the solution, which was subsequently incubated at −20 °C overnight. The solution was then centrifuged at 25,000× *g* for 20 min, after which the supernatant was discarded. The precipitate was sonicated in 200 μL of tetraethylammonium bromide (TEAB; 0.5 M) for 15 min at 200 W and then centrifuged at 25,000× *g* for 20 min, after which the final supernatant was used for quantification and subsequent experiments. Quantification was performed using a Pierce BCA protein assay kit (Thermo Scientific, Waltham, MA, USA).

### 2.4. iTRAQ Protein Digestion, Labeling, and High-Performance Liquid Chromatography Sample Preparation

Protein digestion was performed in accordance with the FASP procedure described previously [29], and the resulting peptide mixture was labeled using the 4-plex/8-plex iTRAQ reagent according to the manufacturer’s instructions (Applied Biosystems, Beijing, China). Briefly, 2 μg of each sample was mixed with 30 μL of STD buffer (4% SDS, 100 mM DTT, 150 mM Tris-HCl, pH 8.0). The detergent, DTT, and other low-molecular-weight components were removed by repeated ultrafiltration (Microcon units, 30 kD) with UA buffer (8 M urea, 150 mM Tris-HCl, pH 8.0). Subsequently, 100 μL of 0.05 M iodoacetamide was added to the UA buffer to block the reduced cysteine residues, and the samples were incubated for 20 min in darkness. The filters were washed with 100 μL of UA buffer three times and then with 100 μL of DS buffer (50 mM triethylammonium bicarbonate at pH 8.5) twice. Finally, the protein suspensions were digested with 40 μL of DS buffer containing 2 μg of trypsin (Promega, Beijing, China) overnight at 37 °C, after which the peptides were collected. The peptide content was estimated at an extinction coefficient of 1.1 in a 0.1% (g/L) solution by UV light spectral density at 280 nm. The extinction coefficient was calculated on the basis of the frequency of tryptophan and tyrosine invertebrate proteins. For labeling, each iTRAQ reagent was dissolved in 70 μL of ethanol and added to the respective peptide mixture. The samples were labeled iTRAQ-116 for the treatment group and iTRAQl-119 for the control group and were multiplexed and vacuum-dried.

### 2.5. Peptide Fractionation with Strong Cation Exchange Chromatography

iTRAQ-labeled peptides were fractionated by strong cation exchange (SCX) chromatography using the AKTA purifier system (GE Healthcare, Chicago, IL, USA). The dried peptide mixture was reconstituted and acidified with 2 mL of buffer A (10 mM KH_2_PO_4_ in 25% acetonitrile [ACN], pH 3.0) and loaded onto a 4.6 × 250 mm column. The peptides were washed at a flow rate of 1 mL/min with a gradient of 0%–5% buffer B (2 M KCl, 10 mM KH_2_PO_4_ in 25% ACN, pH 2.7) for 5 min, 5–10% buffer B for 10–15 min, 10–30% buffer B for 25–35 min, and 30–50% buffer B for 35–50 min. The elution was monitored by absorbance at 214 nm, and the fractions were collected every minute. The tryptic peptides were extracted, and the peptide mixtures were concentrated to dryness by a SpeedVac centrifuge and redissolved in 2% ACN in 0.1% formic acid before liquid chromatography with tandem mass spectrometry (LC–MS/MS) analysis.

### 2.6. LC–MS/MS Analysis

The experiments were performed on a Q Exactive mass spectrometer coupled to an Easy nLC (Proxeon Biosystems, Thermo Fisher Scientific, Waltham, MA, USA); 10 μL of each fraction was injected for nano-LC–MS/MS analysis. The peptide mixture (5 μg) was loaded onto a C18 reversed-phase column (Thermo Scientific Easy Column; length, 10 cm; inner diameter, 75 μm; 3-μm resin) in buffer A (0.1% formic acid) and separated with a linear gradient of buffer B (80% acetonitrile and 0.1% formic acid) at a flow rate of 300 nL/min controlled by IntelliFlow Technology over 140 min. The spray voltage was set to 2.5 kV, and the temperature of the heated capillary was set to 150 °C. MS data were acquired using a data-dependent top 10 method for dynamically choosing the most abundant precursor ions from the survey scan (300–1800 *m*/*z*) for HCD fragmentation. The determination of the target value was based on predictive automatic gain control (pAGC). The dynamic exclusion duration was 60 s. Survey scans were acquired at a resolution of 70,000 at 200 *m*/*z*, and the resolution of the HCD spectra was set to 17,500 at 200 *m*/*z*. The normalized collision energy was 30 eV, and the underfill ratio, which specifies the minimum percentage of the target value likely to be reached at the maximum fill time, was defined as 0.1%. The instrument was run with the peptide recognition mode enabled.

### 2.7. Sequence Database Searching and Data Analysis

The data were analyzed using DATA ANALYSIS 4.0 (Bruker, Billerica, MA, USA). MS/MS spectra were searched using the MASCOT engine (Matrix Science, London, UK; version 2.2) and analyzed by Scaffold software Q+S. On the basis of the relative abundance of different iTRAQ tags, the peptides derived from different groups were quantified by Scaffold software, and the results are presented as the ratio of one group to another. The relative quantification of the protein concentration was calculated by using the relative quantification of the peptide concentration, which is expressed as the average ratio. The relative quantification of the proteins was determined to be statistically significant when the average ratio was greater than 1.5, and the *p* value was less than 0.05. All the results are expressed as the mean ± standard deviation (SD). These data were analyzed using SPSS Statistics 22.0, and *p* < 0.05 was considered to indicate statistical significance.

## 3. Results

### 3.1. iTRAQ-Based Identification and Quantitative Proteomic Analysis of the Pituitary Gland

**Figure 1:**
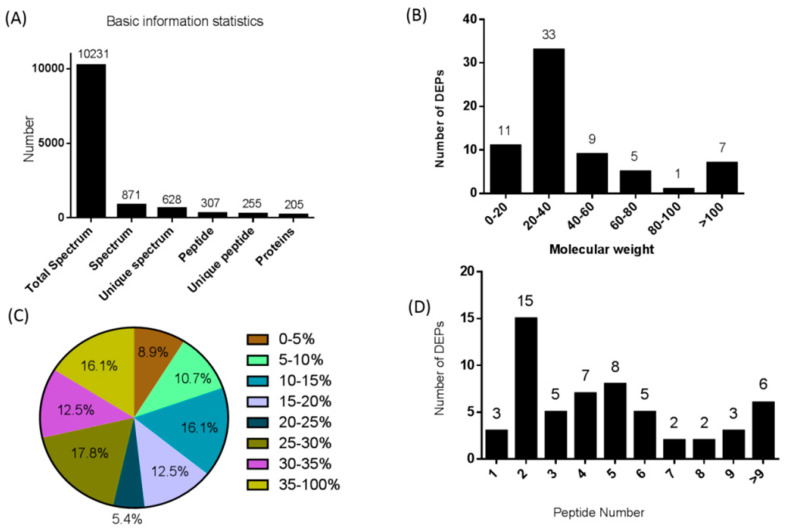
Protein identification and analysis. (**A**) Basic information on the proteins identified. (**B**) Distribution of the DEPs among the different molecular weight classes (in kD). (**C**) Coverage of differentially expressed proteins (DEPs) by the identified peptides. (**D**) Distribution of DEPs containing different numbers of identified peptides.

We used differential isotopic labeling with the iTRAQ reagent to identify differentially expressed proteins (DEPs) in pigs between the heat stress and control groups. All subsequent protein and peptide identifications were obtained by database searching and stringent data filtering. LC–MS/MS analysis produced 10,231 spectra, which corresponded to 255 unique peptides, and 56 proteins were identified at a false discovery rate (FDR) ≤ 0.01 (Figure 1A). We identified 205 DEPs, 31 of which were upregulated and 25 of which were downregulated in the HA-CA group (Table 1).

The molecular weights of the DEPs ranged from 0 to 20 kD (11), 20 to 40 kD (33), 40 to 60 kD (9), 60 to 80 kD (5), 80 to 100 kD (1), or >100 kD (7) (Figure 1B). In addition, the identified DEPs exhibited high peptide coverage, with 85% and 52% showing more than 10% and 20% sequence coverage, respectively (Figure 1C). Approximately 79.65% of the identified DEPs had three or more peptides (Figure 1D).

### 3.2. Subcellular and Functional Characterization of DEPs and Bioinformatics Analysis

**Figure 2:**
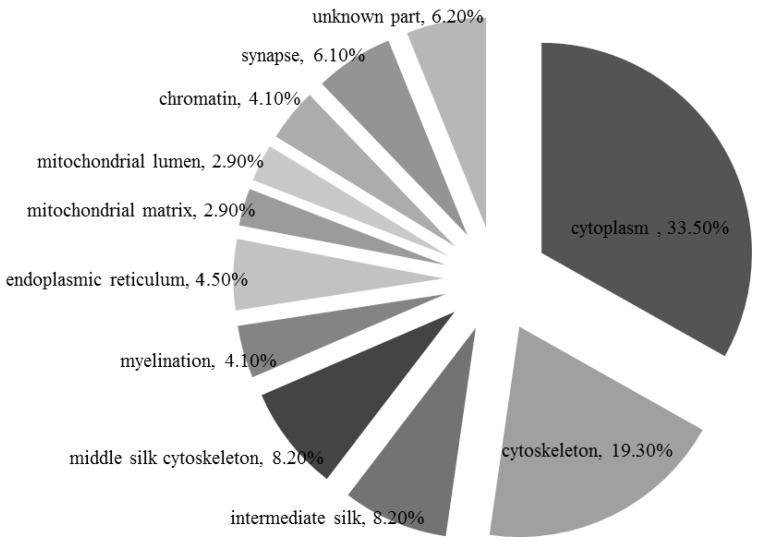
Subcellular locations of the proteins with differential expression (*p* ≤ 0.05) in the HC–CC cohort.

To gain functional insights into the cellular proteome, Gene Ontology (GO) annotation was used to determine the subcellular localization of the 226 DEPs. The 226 DEPs from the pituitary gland under heat stress were localized to the cytoplasm (33.5%), cytoskeleton (19.3%), intermediate silk (8.2%), middle silk cytoskeleton (8.2%), myelination (4.1%), endoplasmic reticulum (4.5%), mitochondrial matrix (2.9%), mitochondrial lumen (2.9%), chromatin (4.1%), synapse (6.1%), and an unknown part (6.2%) (Figure 2).

**Figure 3:**
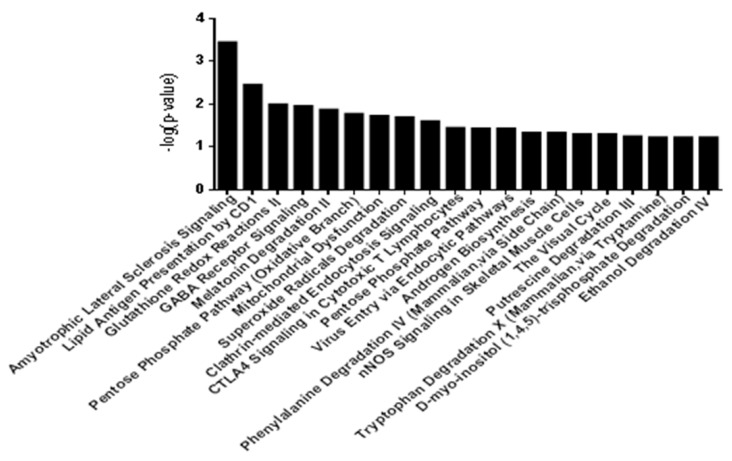
The twenty most related canonical pathways according to IPA.

Since the pig genome database has poorer annotations than does the human genome and because many proteins were unassigned or uncharacterized, the gene identifications of the identified proteins in Table 1 were converted to human protein GeneInfo Identifier (GI) numbers. To better understand these 56 DEPs, we used the Ingenuity Pathway Analysis (IPA) tool for further analysis. Canonical pathways were first examined, and the top 20 pathways are shown in Figure 3. These pathways are associated with inflammation and immunity, including the synthesis and degradation of immune proteins and the conduction of response signals in the acute phase.

The DEPs in the pituitary gland identified by iTRAQ were clustered according to different functions. Four functional groups were identified, which provided meaningful information. Diseases and disorders, molecular and cellular functions, physiological system development, and functions and toxicity functions that were identified at statistically significant levels (*p* ≤ 0.05) are depicted in Figure 3.

**Figure 4:**
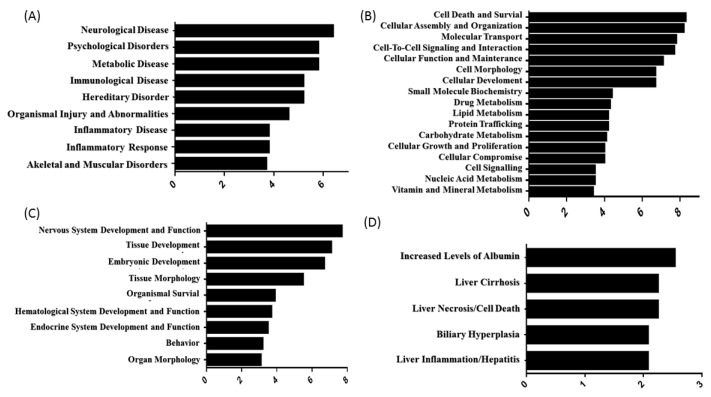
Functional characterization of DEPs in the pituitary gland of heat-stressed pigs. (**A**) Diseases and disorders; (**B**) molecular and cellular functions; (**C**) physiological system development and functions; (**D**) toxicity functions.

The 56 DEPs from the pituitary gland under heat stress, which corresponded to nine diseases and disorders (Figure 4A), included proteins related to neurological disease, psychological disease, metabolic disease, immunological disease, hereditary disorder, organismal injury and abnormalities, inflammatory disease, the inflammatory response, and skeletal and muscular disorders. These DEPs could also be assigned to 17 molecular and cellular function groups (Figure 4B): namely, cell death and survival, cellular assembly and organization, molecular transport, cell-to-cell signaling and interaction, cellular function and maintenance, cell morphology, cellular development, small-molecule biochemistry, drug metabolism, lipid metabolism, protein trafficking, carbohydrate metabolism, cellular growth and proliferation, cellular compromise, cell signaling, nucleic acid metabolism, and vitamin and mineral metabolism; nine physiological system developmental function groups (Figure 4C): namely, nervous system development and function, tissue development, embryonic development, tissue morphology, organismal survival, hematological system development and function, endocrine system development and function, behavior, and organ morphology; and five toxicity function groups (Figure 4D): namely, increased levels of albumin, liver cirrhosis, liver necrosis/cell death, biliary hyperplasia, and liver inflammation/hepatitis.

**Figure 5:**
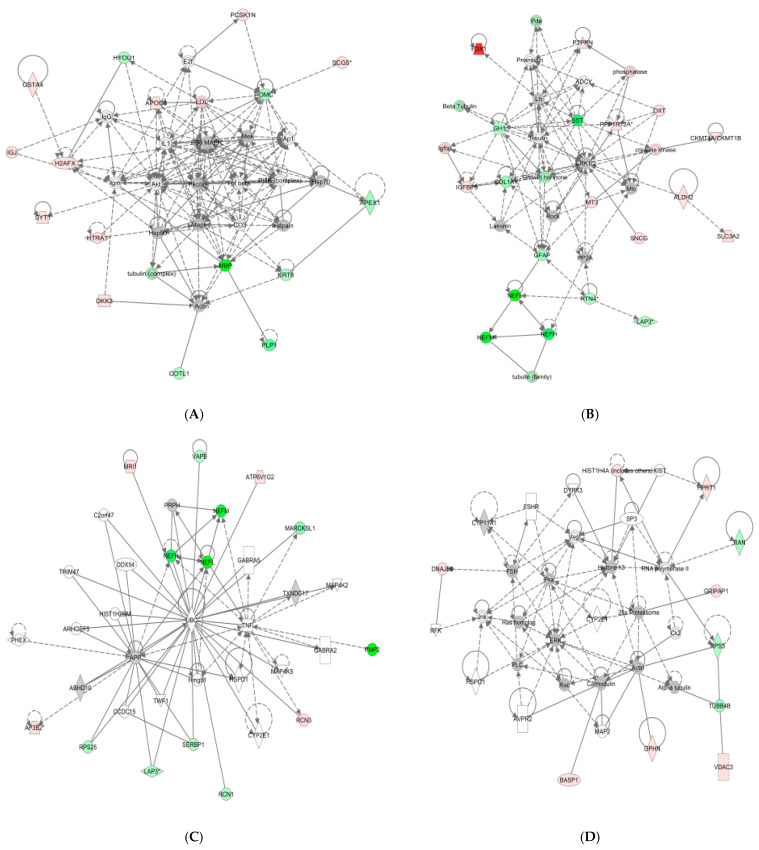
Ingenuity pathway analysis of proteins significantly altered in heat-stressed pigs. Red, upregulated proteins; green, significantly downregulated proteins; white, proteins known to be in the network but not identified in our study. The color indicates the magnitude of the change in the protein expression level. The shapes are indicative of the molecular class (i.e., protein family). Lines connecting the molecules indicate molecular relationships. The dashed lines indicate indirect interactions, and the solid lines indicate direct interactions. The arrow styles indicate specific molecular relationships and the directionality of the interaction. (**A**) Network1: cell assembly and organization. (**B**) Network2: intercellular signals and interactions. (**C**) Network3: Nervous system development and function. (**D**) Network4: Neurological, immunological, and inflammatory diseases.

Proteins whose expression changed significantly in the pituitary gland under heat stress were mapped to the following four functional networks (Figure 5): (1) cell assembly and organization (Figure 5A); (2) intercellular signals and interactions (Figure 5B); (3) nervous system development and function (Figure 5C); (4) neurological diseases, immune diseases, and inflammatory diseases (Figure 5D). Proteins that are present in these pathways and identified as upregulated in our analysis are depicted in shades of red, and those that were identified as downregulated are shown in green. Proteins that are known to be in the network but were not identified in our study are depicted in white. Upstream regulators of the differential pituitary proteins included the pituitary adenylyl cyclase-activating peptide, Ca^2+^, and epidermal growth factor.

## 4. Discussion

Stress leads to the formation of endocrine factors such as glucocorticoids and catecholamines, which can cause immune cell apoptosis, lymphocyte proliferation, and dendritic cell (DC) dysfunction, thereby inducing immunosuppression in animals [19,20]. However, to address the debate regarding the dynamics of glucocorticoid changes under heat stress across various studies, we used iTRAQ technology to identify differentially expressed proteins in the pituitary under heat stress conditions. A total of 56 different proteins involved in intercellular communication, interaction, apoptosis, nervous system development, function, abnormalities, and other functions were identified, as was the regulatory network. Moreover, the upstream regulators of the differentially expressed proteins were mainly transcriptional regulators, hormones, and cytokines. These findings provide theoretical support for systematically revealing the regulatory mechanisms underlying stress-induced immunosuppression.

Ferredoxin (Fd) is an iron–sulfur clustered small-molecule protein widely found in plants, animals, bacteria and fungi, protists, and viruses. It is involved in various electron transport processes in a range of metabolic reactions. Studies have shown that energy transduction in intestinal microorganisms is dependent on membrane-bound Fd [30]. In *Methanobrevibacter woesei* and *Clostridium*, it acts as an electron acceptor for pyruvate-Fd-oxidoreductase (acetyl-CoA formation) [30,31]. Heat stress has been proven to induce disorders of the intestinal flora. He et al. showed that the increase in *Halomonas* and *Methanobrevibacter woesei* may reflect the deterioration of the intestinal environment owing to heat stress [32]. The 31 upregulated proteins in the pig pituitary identified in this study included the Fd protein, which may also be due to the disordered intestinal microflora caused by heat stress, resulting in robust energy metabolism by harmful bacteria such as *Halomonas* and *Methanobrevibacter woesei*. However, further large-scale studies are required to determine the roles and interrelationships of Fd in pigs.

Gephyrin is a scaffolding protein in the dense inhibitory postsynaptic region that anchors and stabilizes GABA-A receptors and glycine receptors on inhibitory synapses. It plays an important role in the formation and function of inhibitory synapses [33]. Machado et al. showed that the synaptic accumulation of gephyrin was significantly decreased upon the overexpression of the heat shock cognate protein 70 (Hsc70) in mouse spinal cord neurons, and Hsc70 inhibition increased gephyrin accumulation at inhibitory synapses [34]. In the present study, gephyrin expression in the pituitary gland was upregulated 2.9-fold during heat stress, disturbing the balance between the excitatory and inhibitory synapses and leading to sudden triggering abnormalities, possibly due to heat damage to animal nerves.

Metallothioneins (MTs) are a class of low-molecular-weight, highly conserved metal-rich and sulfur-containing cytoplasmic proteins that are important for zinc and copper homeostasis, protection against oxidative stress, and buffering against toxic heavy metals [35]. Many studies have revealed the relationship between heat stress and metallothioneins in animals and plants. Luo et al. showed that exogenous metallothionein could enhance anti-heat stress and antioxidant capacity by upregulating the expression levels of SOD, GSH-Px, CAT, HSP70, and the *Bcl-2* gene in the liver tissue of pigs and calves [36]. Zn supplementation can regulate MT expression to alleviate ochratoxin A-induced oxidative stress and DNA damage in HepG2 cells and MDCK cells [37,38]. In the present study, the expression of MT-III was upregulated approximately 2.18-fold among the DEPs in the pituitary gland under heat stress, which suggested that MT-III may be involved in heat stress-induced immunosuppressive reactions and is related to the homeostasis of animals under heat stress.

The myelin basic protein (MBP) is a single-stranded flexible polypeptide located in the dense myelin sheath and nucleus pulposus. MBP plays an important role in assisting neurons in transmitting nerve signals and maintaining the blood–brain barrier. Many studies have confirmed that heat stress can induce changes in blood–brain barrier permeability, the cerebral blood flow, brain edema, and serotonin levels, thereby affecting the regulation of immune function [39,40,41]. Studies have shown a substantial reduction in MBP immunostaining in the brains of heat stress-treated rats [42]. In the present study, compared with that in the control group, MBP expression in the heat stress group was downregulated 0.175-fold, suggesting that heat stress may reduce the expression of MBP, thereby destroying the integrity of the blood–brain barrier and ultimately affecting the regulation of the immune function in the nervous system.

The growth hormone (GH) is a protein hormone secreted by the GH-secreting cells of eosinophilic granules in the anterior pituitary that promotes growth and metabolism and protects the integrity of the nervous system [43]. Parkhie et al. reported that exposure to heat for 1 or 24 h resulted in enhanced hypothalamic GH release in rats, with a concomitant marked depletion of the pituitary GH content [44]. Similarly, Roushdy et al. showed that thermal stress for 6 h downregulated the expression of the *GH* gene in the liver of broilers [45]. In the present study, the expression level of the GH was downregulated 0.601-fold under heat stress. The inhibition of the GH may play a role in the immunosuppression caused by heat stress, not only by inhibiting the metabolism of the body, but also by affecting the development and function of the nervous system. However, further large-scale studies are needed to determine the roles and interrelationships associated with the GH in pigs.

## 5. Conclusions

This study compared the differential protein functional networks of the pituitary gland between healthy controls and pigs exposed to heat stress. Using the iTRAQ technique, 56 DEPs were identified from the porcine pituitary gland under heat stress. The ingenuity pathway analysis (IPA) results indicated that these differentially expressed proteins were involved in intercellular communication, interaction, apoptosis, nervous system development, function, abnormalities, and other functions and in the regulatory network. Moreover, the upstream regulators of the differentially expressed proteins were mainly transcriptional regulators, hormones, and cytokines.

## Figures and Tables

**Table 1:** The proteins significantly changed from the pituitary gland in pigs under heat stress.

Protein Name	Accession Number	Ratio (Heat Stress/ Control)	Peptides	Functions
Downregulated protein in pituitary gland				
RTN4-Aw	gi|38488990	0.666	4	
Cytosol aminopeptidase-like	gi|350587377	0.66	2	manganese ion binding
Plasminogen activator inhibitor 1 RNA-binding protein-like isoform 2	gi|311259195	0.652	3	
Reticulocalbin-1-like isoform 2	gi|350580184	0.642	5	protein binding and calcium ion binding
40S ribosomal protein S25-like isoform 1	gi|311264034	0.629	4	RNA binding and structural constituent of ribosome
Growth hormone	gi|134715	0.601	13	growth hormone receptor binding, ion binding and hormone activity
APEX nuclease 1	gi|210062866	0.555	3	transcription corepressor activity
Collagen alpha-1(I) chain	gi|350590450	0.493	2	
Proteolipid protein	gi|5679718	0.363	6	structural constituent of myelin sheath
Neurofilament heavy polypeptide	gi|311270880	0.237	9	
Protein, myelin basic	gi|224358	0.175	4	structural constituent of myelin sheath
Neurofilament light polypeptide	gi|346716234	0.171	16	protein C-terminus binding
Myelin P2 protein	gi|297307127	0.158	4	cholesterol binding
Upregulated protein in pituitary gland				
Ferredoxin	gi|164449	4.945	5	metal ion binding and electron carrier activity
Oxytocin-neurophysin 1	gi|585553	4.469	5	neurohypophyseal hormone activity and oxytocin receptor binding
Glutathione S-transferase A4	gi|343403759	3.183	1	glutathione transferase activity
Immunoglobulin J chain-like isoform 1	gi|335293621	3.029	1	antigen binding
Gephyrin	gi|343183313	2.924	3	cytoskeletal protein binding and protein complex scaffold
DnaJ homolog subfamily B member 9-like isoform 1	gi|311275610	2.375	2	heat shock protein binding
Protein tyrosine phosphatase, receptor type, N isoform 1	gi|311273106	2.37	4	transmembrane receptor protein tyrosine phosphatase activity
Metallothionein-III	gi|2073002	2.18	2	-
Serine protease HTRA1-like	gi|311271945	2.011	2	serine-type endopeptidase activity
Gamma-synuclein	gi|132269870	1.969	7	-
Putative V-ATPase G subunit	gi|6624727	1.947	2	hydrogen-exporting ATPase activity
Secreted neuronal and endocrine protein	gi|120564449	1.836	5	enzyme inhibitor activity
Ig heavy chain variable VDJ region	gi|558859	1.832	1	-
Methylthioribose-1-phosphate isomerase	gi|311248954	1.822	2	identical protein binding
Brain acid soluble protein 1-like isoform 1	gi|311273514	1.704	9	transcription corepressor activity
Insulin-like growth factor-binding protein 5 precursor	gi|300679422	1.701	6	insulin-like growth factor I binding
GRIP1-associated protein 1	gi|350595677	1.696	8	-
Dickkopf homolog 3	gi|88606665	1.691	7	
Hypoxanthine phosphoribosyltransferase 1	gi|71842219	1.683	5	hypoxanthine phosphoribosyltransferase activity
Creatine kinase U-type, mitochondrial-like isoform 1	gi|311244870	1.675	9	creatine kinase activity
AP-3 complex subunit beta-2	gi|350596485	1.668	2	transporter activity
Voltage-dependent anion channel 3	gi|8745556	1.636	2	porin activity and nucleotide binding
Reticulocalbin-3-like	gi|311257971	1.632	6	calcium ion binding
Histone H2A.x-like	gi|335294990	1.629	3	histone binding and damaged DNA binding
Neuroendocrine protein 7B2	gi|112850	1.628	5	enzyme inhibitor activity and unfolded protein binding
Synaptotagmin-1	gi|350584732	1.6	4	1-phosphatidylinositol binding
Apolipoprotein C-III	gi|164361	1.559	4	phospholipid binding and lipase inhibitor activity
Mitochondrial aldehyde dehydrogenase 2	gi|81295909	1.536	6	aldehyde dehydrogenase [NAD(P)+] activity
Protein phosphatase 1 regulatory subunit 12A	gi|350584736	1.536	2	signal transducer activity
Solute carrier family 3 member 2	gi|171465894	1.521	6	calcium:sodium antiporter activity
Histone H4	gi|51317314	1.508	5	Chromatin structure and dynamics

## Data Availability

Data will be made available upon request.
